# Participant Perspectives of Cognitive Rehabilitation for Type 2 Diabetes: Expectations and Impact

**DOI:** 10.1155/2018/6563457

**Published:** 2018-08-19

**Authors:** Heather E. Cuevas, Alexa K. Stuifbergen, Catherine Ward

**Affiliations:** School of Nursing, The University of Texas at Austin, 1710 Red River, Austin, TX 78701, USA

## Abstract

**Purpose:**

To describe the experiences of people with type 2 diabetes who have completed a comprehensive cognitive rehabilitation intervention.

**Method:**

Nineteen participants with type 2 diabetes enrolled in an 8-week intervention consisting of 4 educational classes to learn strategies to improve cognitive function and an online training program at home to practice cognitively stimulating activities. Two focus groups were conducted as part of a study designed to assess the feasibility of the comprehensive cognitive rehabilitation intervention.

**Results:**

Three main themes were identified in the qualitative data: (1) expectations of cognitive change; (2) use of cognitive strategies; and (3) effect on diabetes self-management. Participants shared valuable insight into how their experiences with the intervention changed and how they viewed diabetes.

**Conclusions:**

While the participants did not initially tie their cognitive complaints to diabetes, they were able to show how and why they might use cognitive strategies to improve diabetes self-management activities. By adapting those strategies for diabetes, quality of life can improve as well as potentially glycemic control.

## 1. Introduction

Older adults with type 2 diabetes (T2DM) are at higher risk for dementia than those without T2DM, and once cognitive problems have been diagnosed, there is a potential for faster progression of cognitive decline [1]. Additionally, cognitive decline may begin early in T2DM's development since a diagnosis of T2DM at midlife is associated with about a 20% greater deterioration in cognitive function over 20 years than for those without T2DM [2]. Attention has been paid to changes in cognitive function in the context of diabetes, but few studies have examined how cognitive function affects diabetes self-management [3, 4]. This is unfortunate because if the ability to complete self-management activities such as dietary management, physical activity, and glucose self-monitoring is altered, then interventions to educate patients may not be successful.

Prior examinations have addressed self-management activities, but focused on only one aspect of diabetes self-management and/or one cognitive domain and have had limited inclusion of underrepresented minority populations [5, 6]. Preliminary investigations have demonstrated connections between lower levels of perceived cognitive function (memory and executive function) and diabetes self-management adherence, but testing of cognitive rehabilitation interventions in diabetes is a novel and understudied focus.

The National Academy of Neuropsychology (NAN) has stated that aspects of effective cognitive rehabilitation include teaching both compensatory skills and brain retraining [7]. Practice and repetition (online computer training) can strengthen skills (e.g., memory, attention, and executive function), and these improvements can be translated into more complex functional behaviors through integration with compensatory strategies that are taught in person [8]. Comprehensive cognitive rehabilitation interventions in other chronic conditions have been shown to be effective in improving function in daily life, but this has not been demonstrated in diabetes [9–12].

Due to this gap, a comprehensive cognitive rehabilitation intervention was developed, and 19 people with T2DM were invited to participate. This project was built on prior work examining the relationships of perceived memory, cognitive ability, and T2DM self-management [13]. One of the aims of the study was to determine the acceptability of this type of intervention in this population. Cognitive training can give individuals a greater sense of control over cognitive changes as well as have a beneficial effect on quality of life [14]. Therefore, central to understanding if these types of investigations will be effective is to explore the experiences of participants who have taken part in comprehensive cognitive rehabilitation interventions.

The purpose of this paper was to describe and focus specifically on the perceptions of people with T2DM in a cognitive rehabilitation intervention in order to refine it for testing in a randomized controlled trial with a larger sample. Qualitative studies have examined participant perceptions of comprehensive cognitive rehabilitation interventions in other chronic conditions. Haesner et al. [15] found that 60 older adults, both with and without mild cognitive impairment, who took part in an computer-based cognitive rehabilitation intervention expressed interest in regular, long-term use of the cognitive training platform that included both social (“chat”) and mental (“brain training” exercises) features and emphasized that the content of the exercises should have a connection to their daily activities.

In a metasynthesis of qualitative research on perceptions of people participating in group-based cognitive rehabilitation, das Nair et al. [16] found that cognitive rehabilitation was associated with increased confidence, self-awareness, and learning of new skills and strategies to compensate for deficits. Additionally, they found that those changes had a positive impact on daily life in personal and professional spheres [16]. das Nair and Lincoln [17] also found that participation in a cognitive rehabilitation intervention improved insight and awareness of memory problems, knowledge, and use of memory aids as well as improved mood and assertiveness. Participants also reported an altered perspective of life that helped them deal with their problems [17]. These themes have been echoed in other analyses of cognitive rehabilitation programs [18–20]. However, qualitative examinations of cognitive rehabilitation interventions for those with type 2 diabetes have not been done. Further research is needed in light of the effect the interventions have on confidence and acquisition of new skills—concepts that are strongly linked to self-management of type 2 diabetes [21].

## 2. Methodology

### 2.1. Design

This qualitative descriptive study was part of a project (Clinical Trials Registration NCT03221452) to adapt a comprehensive cognitive rehabilitation intervention originally designed for multiple sclerosis for people with T2DM. The primary aim of the main study was to adapt and test the feasibility of the intervention in a population (people with T2DM) for which it was not originally designed.

The intervention consisted of 4 2-hour educational sessions held every other week to teach compensatory cognitive strategies. Participants were also asked to complete 45 minutes of an online brain-training program three times a week at home. All class sessions emphasized maximizing cognitive function first and then addressed the interrelated effects of improved cognition on self-management skills and improved glycemic control on cognition. Each class session followed the same basic format: (a) introduction/revisiting content from the prior class and answering questions; (b) review of progress on computer exercises prescribed in the previous class; (c) practice of cognitive strategies in the class; (d) content on weekly topic; and (e) closure—prescribed computer training and cognitive strategy assignments for the upcoming week. Discussion on how to use this information in day-to-day self-management activities was also included. Participants received notebooks with class content to support what was taught in each session.  Table 1 includes a description of each of the classes.

For both the feasibility study and the qualitative substudy, university ethics approval was obtained, and all study participants provided written informed consent.

### 2.2. Participants

Participants were 40 to 70 years old with T2DM living in Central Texas. Demographics are described in Table 2. All participants from the main study were invited to participate in the qualitative substudy.

### 2.3. Data Collection

A trained facilitator, who was not involved in the intervention delivery, completed 2 focus groups of approximately 45 minutes each with 11 participants in one group and 8 participants in the other. The focus groups took place after the last class session of the intervention. Focus group participants were asked about their experiences in the program and cognitive strategies they found most useful as well as motivation and self-efficacy related to use of the cognitive strategies (Table 3). The focus group facilitator took extensive notes and audio-recorded the sessions. The audio recordings were transcribed verbatim. All data were deidentified, and numbers were used to differentiate participant responses (e.g., Participant 1 said…).

### 2.4. Data Analysis

Since this study used a qualitative descriptive design, Miles, Huberman, and Saldana's recommendations for content analysis were employed to review and code the transcripts [22]. In a qualitative descriptive inquiry, little inference is applied to explain information in everyday language so the codes and themes are derived from the data rather than from a theoretical framework [23]. Additionally, conventional content analysis is used to describe a phenomenon when existing research is limited [24]. This approach allows categories to emerge from the data and to use terms that reflect the participants' frameworks and are meaningful to them.

First, the data were read repeatedly to obtain a sense of the whole; next, the data were read word-for-word, highlighting the text that appeared to capture key thoughts and concepts; codes were then derived from the key concepts; and the codes were categorized according to their relationships and linked and grouped into meaningful clusters [22]. A second member of the research team separately coded the transcripts using the themes from the initial coding. Agreement between the two coders was 90%. In the case of disagreements, the items were discussed, and the section of the text was either placed in a different coding category or a new code was created from similar themes. During coding, notes were made on the procedures, analysis, and reflections on the emerging themes.

## 3. Results

Three main themes were found to describe participants' experiences: (1) expectations of cognitive change; (2) use of cognitive strategies; and (3) effect on diabetes self-management (Table 4). The themes span the participants' experiences with cognitive issues and diabetes self-management from prior to the intervention, during the intervention, and after the intervention was completed.

### 3.1. Expectations of Cognitive Change

Participants were asked to describe how they felt the class sessions and computer training met their expectations as well as their thoughts on what could be changed to make the intervention better. None of the participants expected to have “major improvements in brain function.” Instead, most felt they would be able to learn strategies and put them into practice and improvement would take time that extended beyond taking part in the intervention. Even though most participants did not complete the full time requested for the computer training (averaged 106 minutes out of a requested 135 minutes per week), they had a strong interest in the classes and recommended a continued focus on teaching cognitive strategies “to make life easier.” Several participants said they did not know what to expect from the classes because they had problems obtaining information about cognitive function and diabetes from other sources; for example, “I feel like I was not sure what kinds of questions to ask in the class. No one has ever talked to me before about how diabetes could change how well I think.”

The source and type of information that came from the intervention played a role in what they expected would help their cognition. All said they appreciated clarification on popular “brain games” and information on currently available evidence regarding what the games did and did not do. Some stated this was contrary to the information presented by the press and advertising. One participant commented, “It's hard to know what I need to do when the information and advertising is misleading. And I get that there's still more research that's needed. There's so much out that trying to sell me things. It helped to have someone in health care clear it up for us.” Participants also appreciated explanations of the causes of cognitive problems. Some felt changes in cognitive function were a “slippery slope” to Alzheimer's disease and that nothing could be done to prevent that progress. Because of this, they believed there was no need to make their health-care providers aware of cognitive issues. However, due to the information provided in the intervention, they realized there might be ways to maximize their cognitive function. They now had questions for their health-care providers such as checking for vitamin B12 deficiency related to metformin use or tests that would be useful in investigating other causes of cognitive problems.

All expected to “learn cognitive strategies from each other and share ideas” and preferred a group format. They were split on if the classes should be online (via a program like Skype) or in person in a clinic setting. Regardless of the method, the sharing of strategies to assist cognitive function was a prominent concept brought to light by almost all of the participants. For example, one woman stated, “I wasn't sure what would work and what wouldn't. I didn't anticipate that most people had tried different things, and I want to continue talking about this with others.”

### 3.2. Use of Cognitive Strategies

Most of the participants talked about the difficulties of living with diabetes and perceived cognitive problems. Some were aware of cognitive strategies before the intervention but had not tried them because they felt like “this was just a normal part of getting old.” Others had no prior knowledge of cognitive strategies. All participants tried at least 3 strategies during the 8-week period. Some used a combination of strategies for single tasks. For example, one participant described strategies she used in preparing meals for her family, “It takes a lot. If I think of everything I have to do all at once, I know I forget things. So I now I make a list of groceries and then I quit checking my phone while I'm at [grocery store].” This demonstrated use of planning ahead, list making, and limiting multitasking—all strategies that were discussed in the session on boosting attention. Others were concerned with slow speed of processing and mentioned they would “stay quiet because people will think I'm stupid if I can't come up with an answer quickly.” One person said he started asking people to let him read an item in question (he was a customer service representative). This gave him more time to process the information as well as let him review the concepts discussed.

Challenges of using cognitive strategies were also elucidated, and barriers of initiating planned activities such as an exercise program were prominent findings. For example, “I have every intention to exercise. I make time in my schedule, but something always comes up.” Goal setting assisted with those problems, but a few felt it was not enough: “I need more than just writing down one goal. Sometimes I never look at it again.”

Difficulties in changing habits were noted. Lack of time, lack of “discipline,” and other priorities were barriers to use the online computer training. Another said that “one more thing is being asked of me and I do not know if I can do any more.” Motivation to use the strategies included encouragement from health-care providers. For example, “When I see my physician, she tells me to keep trying to be healthy and to keep us the good work.” Or “I realized that if I don't want to get worse diabetes then I have to take control of my sugars. But I can't always remember what I need to do next. These classes helped me figure out ways to think about what I'm doing and then make a list or set my phone alarm. It's motivation to be think in better ways so my diabetes can be better…and my diabetes nurse can help me.”

Most had a sense of achievement even with small changes such as making lists. They felt the intervention helped increase their “mental abilities” and flexibility with cognitive strategy plans. Others said some of the at-home assignments were too easy and recommended having them increase in difficulty as they progressed through the program. Some wanted more guidance and practice with “real-life” situations that could occur in self-management and suggested text messaging as a way to communicate with the intervention facilitator.

### 3.3. Impact on Diabetes Self-Management

The groups consistently stressed the effect of cognitive problems had on diabetes self-management. Many said, “I forget too easily” or “I have problems planning, I can't think that far ahead.” Most were appreciative of the information related to cognitive changes in diabetes and said the information lessened their concerns fear of Alzheimer's disease.

The cognitive strategies were also felt to be useful in assisting with diabetes self-management, and while some felt the intervention did not have a direct effect on their actual glucose readings, it gave them a greater sense of control and self-efficacy. However, two participants shared their glucose records with the focus group. They believed the cognitive strategies they employed such as list making, avoiding multitasking, and setting specific goals helped with diabetes self-management and improved their glycemic control. One said the meditation practice taught in a session helped to “decrease my anxiety and let me think more clearly about what I needed to do that day. I think anxiety was a big piece of what was keeping me from working on my diabetes and I didn't realize it before.” This sentiment was echoed by 3 others in the group, and they recommended more emphasis on practices such as meditation and deep breathing to help improve focus and decrease the anxiety they associated with diabetes management.

Many described how the intervention helped them look at diabetes self-management through a different lens and think more broadly about the ways good self-management could improve glucose, cholesterol, blood pressure, and cognitive function. Some described the intervention as having short-term and long-term outcomes. Short-term outcomes were related to specific tasks such as the online games and weekly practice of cognitive strategies. The long-term aims were related to more general changes, for example, improving A1C values and “keeping my brain healthy for a longer time.” One participant said it this way: “The shorter goals were things that I knew I needed to do, but I had to keep in mind that they were for a bigger purpose. I need to keep doing these things and make them habits so I can be healthier.” Another said: “This isn't the solution to all my diabetes and thinking problems. But it's a good foundation course that I think underlies a lot of other approaches to getting me to work more on thinking better and keeping my glucose controlled.”

## 4. Discussion

### 4.1. Summary

Diabetes is a serious chronic illness that has implications for multiple facets of a person's health, including cognitive function. The theme of “impact on diabetes” emphasizes participants' experiences with self-management in light of any perceived cognitive problems. Participants often described not meeting the self-management standards that were set for them (by themselves or by their health-care provider) and felt that anything they could do to improve cognitive function would also help with diabetes self-management. The relationship of perceived cognitive function and diabetes self-management has been seen in the recent literature [13, 25] and suggests perceived executive function and memory capabilities are significantly associated with diabetes self-management adherence. Other studies have also found that self-management and glycemic outcomes are even worse when screening for cognitive dysfunction is delayed despite complaints of cognitive problems [26]. This indicates a positive relationship and involvement with health-care professionals are essential [27]. However, like this group of participants, many people attribute the changes in cognitive function to “old age” or to “natural processes” and are misinformed about activities that can support cognitive health—both within and outside of the context of diabetes—such as diet, exercise, and blood pressure control. Based on this study's findings, Table 5 lists recommended features for cognitive rehabilitation interventions, which are discussed in more detail below.

### 4.2. Expectations of Cognitive Change

Participants in comprehensive cognitive rehabilitation interventions have shown improvement on neuropsychological tests and have reported the interventions' cognitive strategies that improved their ability to function in daily life [12, 28–30]. Therefore, testing interventions of this type shows promise for people with diabetes. As results of this study demonstrate, many people with T2DM are unaware of not only the interventions that may benefit cognitive function, but also lack information on the effect diabetes can have on cognitive function.

This issue highlights a need for increased education and counseling on diabetes self-management, the risk of cognitive dysfunction, and the expected benefits of cognitive rehabilitation. The use of tested interventions is important due to the marketing efforts of certain “brain games” which consumers may interpret as cures for cognitive problems or that games will definitively prevent diseases such as Alzheimer's. Using an educational approach that incorporates goal setting along with practice of realistic, practical activities allows people to manage their expectations and empower them to increase adherence to their diabetes self-management plan. Furthermore, heightened understanding of the persons' experiences with these interventions may further increase the capacity of health-care providers to address questions patients do not know to ask and improve the probability that they receive accurate, evidence-based recommendations.

In this study, one person stopped participating after one class. He felt that the class was not going to give him the type of progress he wanted (he wanted something that would “work faster”). This is in line with behavior change theory that finds interventions are discontinued when expectations do not match results [31]. In cases such as this, it may be helpful to emphasize the aspects of the intervention that improve both global cognition and glycemic control, such as physical exercise. Stressing the potentially additive benefits of a multimodal approach may improve retention as well as outcomes [32].

### 4.3. Use of Cognitive Strategies

Consistent with previous research [9, 28, 29], the most common barriers for using the strategies learned in the intervention were time and low motivation. A meta-analysis of adherence in cognitive rehabilitation studies showed adherence to the interventions ranged from 62% to 67% [33], but in this study, most participants did not fulfill the full time requested for the online computer training. Some felt the exercises were not making a difference and wanted to focus on the “useful hands-on strategies” instead. Indeed, most of the participants felt their time was better spent practicing the cognitive strategies taught in the class. Participants also said that life events (work and family demands) sometimes caused them to miss practicing the weeks' activities. But most indicated that they tried to make up the time or practice the strategies at a later time. Some participants were initially frustrated with the online portion of the intervention due to difficulty or confusion with the instructions, which lowered motivation levels. They felt the time on the online training reported by the program was inaccurate and did not account for the time spent reading directions and trying to understand how to play the games.

In the future, it may be beneficial to have one-on-one training with a facilitator to help them overcome those initial problems. It is also possible that contact with a health-care provider who has “prescribed” cognitive rehabilitation may decrease anxiety and motivate patients to meet the recommended practice times [34–36]. Motivational interviewing strategies have been effective in increasing adherence to cognitive rehabilitation interventions in other chronic conditions [37–39] and could be useful in diabetes. By integrating motivational interviewing principles into the intervention, intervention facilitators can investigate and address any ambivalence or emotional factors participants may have and possibly increase adherence.

### 4.4. Impact on Diabetes Self-Management

Baseline adherence to diabetes self-management activities was low, and the focus group results show that knowledge was a barrier to diabetes self-management changes. This again highlights the need for public health information regarding diabetes and cognitive health. The risks are there, but it is something that may or may not be discussed with health-care providers. Incorrect information can have an impact of whether or not to make lifestyle changes. Health-care professionals are often the contact point for clarifying health information and are an essential source for those concerned about cognitive problems in the context of diabetes self-management.

Participants frequently mentioned goal setting as one helpful strategy, but they wanted guidance from health-care professionals in setting and monitoring goals. This has implications for adherence since goal setting has been shown to be a part of effective diabetes self-management and cognitive rehabilitation [40, 41]. The selection of goals by participants can create more meaning, maximize outcomes, and increase adherence. For example, Rodakowski et al. [42] found that older adults with mild cognitive impairment in a cognitive rehabilitation program valued self-selection of exercise goals. Additionally, Lie et al. [43] and D. B. Reuben and M. E. Tinetti [44] found that combining a goal-directed diabetes eHealth intervention with face-to-face consultations had potential to increase motivation and adherence. The results here indicate goal setting using “real-world” situations was critical and added to diabetes self-management adherence.

Participants feared developing dementia and worsening of any perceived cognitive problems. These findings are consistent with Kim et al. [45] who found in their investigation of glucose variability and cognitive function that a majority of their participants had the same worries. In this project, feedback from the class facilitator and other participants was clearly a motivator for trying new cognitive strategies and completing the brain-training homework. But while feedback indicated that the social component of the intervention was valuable to discuss common self-management concerns, the intervention required time and sometimes time off from work. The authors believe this was the main problem with recruitment and retention. It was challenging to recruit participants, and it took 8 weeks to enroll the first participant. At that point, modifications were made to the class schedule to change it from weekly classes over 8 weeks to 4 classes every other week for 8 weeks.

Alternatives to the intervention format suggested by the focus groups included online/virtually based classes or phone conferencing to reduce the time and travel burden.

Studies have demonstrated that diabetes self-management education can be effectively delivered via technology such as online conferencing and can be effective in improving both physiologic and behavioral outcomes [46]. Patients who have gone through such programs have reported more satisfaction with access to the provider as well as decreasing the time and travel burden associated with attending classes [47]. However, the research on cognitive rehabilitation using distance learning format is sparse in the context of chronic illnesses. A meta-analysis in 2014 showed distance learning alone did not produce improvement. Improvements were only seen in those people who were supervised by a trainer in 1 to 3 sessions per week [48]. The authors of the meta-analysis also noted that the findings were only specific to healthy older adults and may not apply to those who already have chronic illness, and future research should explore these methods [48]. In this project, the social contact supported self-management behavior change and discussion with other participants that provided aspects of role modeling that should be included in future iterations of the intervention.

#### 4.4.1. Limitations

One of the aims of this study was to test the acceptability of a comprehensive cognitive rehabilitation intervention for people with T2DM. While a good description of the perceptions of the intervention was obtained from the participants, there are several limitations. The first is that the results are limited by convenience sampling. It is possible that purposive sampling would give a broader range of views than what was found in this project. Also participation may reflect motivation for behavioral change that may not be present in the general population with perceived cognitive problems and/or diabetes. However, this is the first study, to our knowledge, examining a comprehensive cognitive rehabilitation intervention to deal with both conditions.

#### 4.4.2. Clinical Implications

While participants in this project did not originally tie their cognitive concerns to diabetes, they showed how and why they used cognitive strategies to enhance diabetes self-management activities by the end of the intervention. Currently, there are no standardized treatment recommendations for cognitive function in the context of diabetes, but the results here demonstrate that comprehensive cognitive rehabilitation interventions may be promising. In adapting the strategies for diabetes, there is a potential for improved quality of life. Future research needs to identify barriers to cognitive activities and strategy use, such as the time and emotional factors found here, which may be important to assist in self-management and therefore glycemic control.

## Figures and Tables

**Table 1:** Class content.

	Class content
Week 1	(i) Understanding T2DM, symptoms, complications, and medications.
	(ii) Understanding how cognitive function is related to T2DM.
	(iii) Orientation to computer training.
	(iv) Discussing effective strategies to facilitate better communication with health-care providers, for example, understanding instructions or recommendations from health-care providers.
	(v) Strategies to enhance attention and problem solving.

Week 3	(i) Strategies to enhance memory.
	(ii) Addressing resources and barriers to self-management (e.g., planning ahead for meals and organizing medications) that take into account elements of executive functioning.
	(iii) Visuospatial skills required for blood glucose self-monitoring.

Week 5	(i) Addressing ADA dietary recommendations and how they can benefit cognitive health.
	(ii) Discussion of favorite recipes, more healthy food preparation, eating out, and emphasis on portion control.
	(iii) Acknowledging and appreciating stress associated with diabetes and cognitive issues.
	(iv) Providing resources for mental health-care services.
	(v) Strategies to manage stress.

Week 8	(i) Addressing ADA activity recommendations and benefits of following the guidelines on cognitive function.
	(ii) Discussion of practical ways to increase activity.
	(iii) Review of cognitive skills/training and the potential impact on self-management skills including blood glucose monitoring, medication adherence, diet, and exercise.
	(iv) Addressing resources and barriers to maintaining cognitive function.

**Table 2:** Participant characteristics.

Characteristic	*n*	Range	%	M (SD)
Age in years	19	40–70	—	55.1 (10.9)
Hemoglobin A1C	19	5.4–12	—	8.3 (1.8)
Length of time with T2DM in years	19	2–21	—	7.1 (4.8)
Hispanic	10	—	52.6	—
Non-Hispanic White	6	—	31.6	—
African American	3	—	15.8	—
Female	11	—	57.9	—

T2DM, type 2 diabetes; M, mean; SD, standard deviation.

**Table 3:** Focus group guide.

Cognitive strategies used	Intervention content	Effect of the intervention
What strategies did you try?	What was the most useful thing you learned?	Why did you choose to take part in this project?

Why did you choose those particular strategies?	What could be changed about the classes to make them better?	Have you talked with your health care provider about cognitive issues? How will you approach the topic of cognitive function with your health care provider in the future?

What was difficult?	How did the online sessions go?	What did you know about cognitive rehabilitation before the intervention? Have your ideas about it changed? If so, how?

What worked well?	How will you use the information you learned in the future?	How do you think participating in this project will affect the way you manage diabetes?

**Table 4:** Exemplar quotes.

Themes	Quotes
Expectations of cognitive change	“I wanted my brain to change in a better way. And I think this may be one way to do it. It takes a lot of work, but I think with exercise, diet, paying attention to my sugar and practicing strategies, I can at least help my brain not get worse.”
	“I'm glad to do something like this. I see ads on TV all the time for vitamins for my brain, but I don't know what works. This helps a lot and makes me think better. I like sharing with other people what I work and then I can go to my doctor too and talk about what I've been doing.”

Use of cognitive strategies	“I had no idea I could do things to help my brain. Not all the cognitive strategies are things I've been able to do, but I feel like I'm making small changes and they are super useful.”
	“I think the computer training was fun, but I like working on the cognitive strategies better. I spent more time on making lists and stopping to think things through than on video games.”

Effect on diabetes self-management	“I'm more motivated to work on my diabetes now. I feel like I can do things that will help both my brain and my diabetes at the same time.”
	“I realized that setting small goals will help diabetes and thinking about how adding may be 15 minutes of exercise here and there does a lot—it makes me feel better, it helps my sugar, and it can help me think better.”

**Table 5:** Recommended cognitive rehabilitation intervention features.

Themes	Intervention features
Expectations of cognitive change	(i) Include education on diabetes self-management, cognitive dysfunction, and expected/realistic benefits of cognitive rehabilitation.
	(ii) Use a group format to increase sharing of information related to strategies that may or may not work.
	(iii) Teach participants to ask questions of their health-care providers regarding cognitive function and diabetes.

Use of cognitive strategies	(i) Relate cognitive strategies to everyday activities and/or diabetes self-management tasks.
	(ii) Consider one-on-one training or more frequent contact with a health-care provider familiar with cognitive rehabilitation to facilitate use of online games.
	(iii) Incorporate motivational interviewing techniques to increase adherence and decrease anxiety.

Effect on diabetes self-management	(i) Include feedback from a class facilitator to enhance motivation to complete homework items.
	(ii) Include participants in goal setting using real-world situations as examples.
	(iii) Emphasize the potential benefits of cognitive rehabilitation on both diabetes and cognitive function.
	(iv) Explore alternatives such as providing class instruction via an online meeting format to reduce travel/time burden.

## Data Availability

The data used to support the findings of this study are available from the corresponding author upon request.
